# Reliable and Sensitive Detection of Carbonylated Proteins by Oxime Blot

**DOI:** 10.21769/BioProtoc.5401

**Published:** 2025-08-05

**Authors:** Filip Luka Mikulić, Viktor Merćep, Marcela Finek, Mladen Merćep

**Affiliations:** 1 Department of Emergency Medicine of the Krapina-Zagorje County, Krapina, Croatia; 2 School of Medicine, University of Zagreb, Zagreb, Croatia; 3 Faculty of Biotechnology and Drug Development, University of Rijeka, Rijeka, Croatia; 4 Zora Foundation, Split, Croatia

**Keywords:** Oxidative stress, Carbonylated proteins’ western blot, Oxime blot, 2, 4 dinitrophenylhydrazine (DNPH), Biotin-aminooxy, Age-related diseases, Alpha-synuclein, Parkinson’s disease

## Abstract

Oxidative protein damage is important in various biological processes and age-related diseases. Protein carbonylation is the predominant and most frequently studied form of protein oxidation. It is most frequently detected following its derivatization with 2,4-dinitrophenylhydrazine (DNPH) hapten, followed by its detection with an anti-DNP antibody. However, when used to detect protein carbonylation by western blotting, this method suffers from diminished sensitivity, distortion of protein migration patterns, and unsatisfactory representation of low-abundance proteins. This is due to the poor solubility of DNPH in typical buffer solutions, the acidic protein precipitation due to the use of strong acid for its dissolution, the instability in solution, and the distorted protein migration patterns introduced by an additional salt content generated by the required pH adjustment prior to sodium dodecyl sulfate-polyacrylamide gel electrophoresis (SDS-PAGE). To address the DNPH method limitations, a new Oxime blot technique was developed. This method is based on forming the stable oxime bonds between the protein carbonyl groups and biotin-aminooxy probe in the presence of a p-phenylenediamine (pPDA) catalyst at neutral pH conditions. The derivatization reaction reaches a plateau within 3 h. It ensures efficient and complete derivatization of carbonylated proteins, which are separated by SDS-PAGE without additional manipulation and detected with avidin-HRP and enhanced chemiluminescence (ECL) in western blotting. The Oxime blot protocol allows researchers to reliably and sensitively detect carbonylated proteins and provides a valuable tool for studying oxidative stress in diverse biological settings.

Key features

• This method enables the sensitive and reliable detection of protein carbonylation in various biological samples.

• The chemically stable oxime bond forms quickly and efficiently, reaching its plateau level after 3 h, enabling relative carbonylation quantification.

• Carbonylation derivatization at low salt content and neutral pH ensures good SDS-PAGE protein migration without any protein loss.

• This method integrates well with detecting specific protein carbonylation following its immunoprecipitation.

## Graphical overview


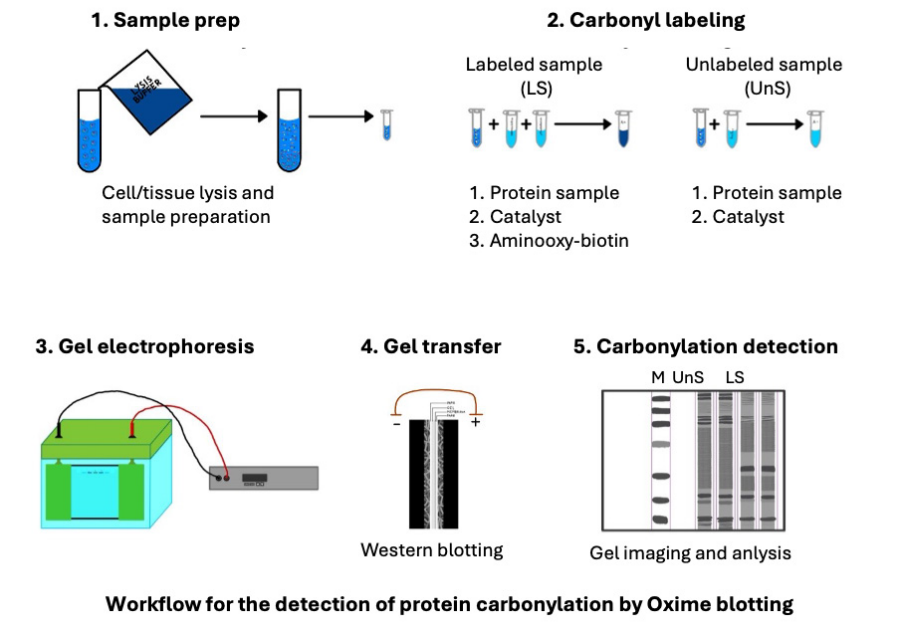


## Background

Reversible protein oxidation is essential in the redox signaling pathways that regulate various physiological processes [1,2]. However, irreversible protein oxidation is much more prevalent. It could impair protein functions and contribute to cellular damage, aging, and age-related diseases such as cardiovascular, neurodegenerative, autoimmune diseases, cancer, cataracts, and type 2 diabetes. It occurs when antioxidant defense systems fail to neutralize the reactive oxygen species (ROS) generated during normal oxidative metabolism or under oxidative stress conditions. This stress can arise from endogenous factors (e.g., inflammation, ischemia-reperfusion injury) or exogenous factors (e.g., radiation, smoking, pollution, psychological stress). There are several forms of oxidative protein damage. The most prominent and widely studied form is protein carbonylation. This irreversible oxidative modification affects amino acid side chains or protein backbones [3–6].

Accurately detecting and measuring protein carbonylation is essential to understanding the role of protein carbonylation in cell biology and age-related diseases [7,8]. The most widely used method relies on carbonyl derivatization with 2,4-dinitrophenylhydrazine (DNPH). The resulting bioconjugate is typically detected with an anti-DNP antibody in enzyme-linked immunosorbent assay (ELISA) or a blot analysis [9,10]. However, DNPH methods have several challenges, such as poor reagent solubility, instability in solution, limited derivatization of carbonyl groups, inadequate representation of the low-abundance proteins, acidic precipitation-induced protein loss, and the need to adjust the pH prior to SDS-PAGE analysis, which could distort protein migration patterns due to the increased salt content. These issues can lead to questionable reliability and reproducibility [10–12].

We developed an alternative method to address these limitations using an aminooxy probe that forms stable oxime bonds with carbonyl groups. This reaction is accelerated by the catalyst p-phenylenediamine (pPDA) and is conducted at neutral pH [13–15]. The carbonyl derivatization with aminooxy reaches the reaction plateau within 3 h, enabling sensitive and reliable carbonylated protein detection. We named this detection method the Oxime blot method [16]. This protocol describes a step-by-step approach to detecting the carbonylation of purified protein and proteins from complex samples such as cell lysates. This protocol has the potential to facilitate more reliable and frequent investigations of protein carbonylation in various cell biology processes and the molecular mechanisms of diseases.

## Materials and reagents

**Biological materials**

1. A549 cell line (ATCC, catalog number: CRL-1555)

**Reagents**

1. Dulbecco’s modified Eagle’s medium (DMEM), high glucose (Sigma-Aldrich, catalog number: D6429)

2. L-glutamine (200 mM) (Pan Biotech, catalog number: P04-80100)

3. Penicillin-streptomycin (5,000 U/mL) (Pan Biotech, catalog number: P06-07100)

4. Fetal bovine serum (FBS) (Pan Biotech, catalog number: P30-3306)

5. Trypsin EDTA solution (Pan Biotech, catalog number: P10-020100)

6. Dulbecco’s phosphate-buffered saline (DPBS) (Pan Biotech, catalog number: P04-35500)

7. Tris base (Sigma-Aldrich, catalog number: 252859)

8. cOmplete^TM^ Mini, EDTA-free protease inhibitor cocktail tablets (Sigma-Aldrich, catalog number: 11836170001)

9. Glycine (Sigma-Aldrich, catalog number: 33226)

10. Sodium dodecyl sulfate (SDS) (Carl Roth, catalog number: 0183.2)

11. Tween^®^-20 (Sigma-Aldrich, catalog number: P-1379)

12. NP-40 as TERGITOL^TM^ solution (Sigma-Aldrich, catalog number: NP40S)

13. Sodium deoxycholate (Roth, catalog number: 3484.2)

14. Aprotinin (Sigma-Aldrich, catalog number: A3428)

15. Leupeptin (Sigma-Aldrich, catalog number: L0649)

16. Sodium fluoride (NaF) (Sigma-Aldrich, catalog number: S1504)

17. Sodium vanadate (Na_3_VO_4_) (Sigma-Aldrich, catalog number: S6508)

18. Phenylmethylsulfonyl fluoride (PMSF) (Sigma-Aldrich, catalog number: P7626)

19. β-Glycerophosphate (β-GP) (Sigma-Aldrich, catalog number: G9422)

20. Sodium dihydrogen phosphate monohydrate (NaH_2_PO_4_·H_2_O) (Sigma-Aldrich, catalog number: S9638)

21. Disodium hydrogen phosphate heptahydrate (Na_2_HPO_4_·7H_2_O) (Sigma-Aldrich, catalog number: S9390)

22. Ethylenediaminetetraacetic acid disodium salt dihydrate (EDTA-Na_2_) (Sigma-Aldrich, catalog number: E4884)

23. Buffer solution 4.01 (Mettler Toledo, catalog number: 51350004)

24. Buffer solution 7.00 (Mettler Toledo, catalog number: 51350006)

25. Buffer solution 9.21 (Mettler Toledo, catalog number: 51350008)

26. 1,4-Dithiothreitol (DTT) (Roche, catalog number: 10197777001)

27. EZ-link alkoxyamine-PEG4-biotin (VWR, catalog number: PI26137)

28. p-Phenylenediamine (pPDA) (Sigma-Aldrich, catalog number: P6001)

29. Dimethyl sulfoxide (DMSO) (Sigma-Aldrich, catalog number: D8418)

30. Resolving gel buffer (Bio-Rad, catalog number: 1619798)

31. Stacking gel buffer (Bio-Rad, catalog number: 1619799)

32. 2-Mercaptoethanol (Carl Roth, catalog number: 4227.3)

33. Glycerol (Kemika, catalog number: 0711901)

34. 3-Color prestained protein marker (10–190 kDa) (MCE MedChemExpress, catalog number: HY-K1011), or equivalent prestained protein ladder

35. 30% Acrylamide-bis solution (Bio-Rad, catalog number: 1610156)

36. N,N,N’,N’-Tetramethyl ethylenediamine (TEMED) (Bio-Rad, catalog number: 161-0800)

37. Ammonium persulfate (APS) (Carl Roth, catalog number: 9178.3)

38. Methanol (Merck KGaA, catalog number: 1060121000)

39. Glacial acetic acid (Honeywell, catalog number: 33209)

40. InstantBlue Coomassie protein stain (Abcam, catalog number: ab119211)

41. Ponceau S (Thermo Scientific, catalog number: J60744.09)

42. Pierce ECL western blotting substrate (Thermo Scientific, catalog number: 32209)

43. Streptavidin, HRP conjugate (EMD Millipore, catalog number: 18-152)

44. Nonfat dry milk (Dukat)

45. Sodium chloride (NaCl) (Fisher Scientific, catalog number: S271500)

46. Bovine serum albumin (BSA) (Capricorn scientific, catalog number: BSA-1S)

47. Bromophenol blue (Sigma-Aldrich, catalog number: B0126)

48. Sodium hydroxide (NaOH) (Sigma-Aldrich, catalog number: S5881)

49. Hydrochloric acid (HCl) (Sigma-Aldrich, catalog number: 258148)

50. Isopropanol (Sigma-Aldrich, catalog number: 34863)

**Solutions**

1. Cell culture medium (see Recipes)

2. 0.1 M phosphate buffer, pH 7.4 (see Recipes)

3. 5 M NaCl (see Recipes)

4. RIPA phosphate lysis buffer with protease and phosphatase inhibitors (see Recipes)

5. 200 mM DTT (see Recipes)

6. 500 mM pPDA with 5 mM DTT (see Recipes)

7. 100 mM biotin-aminooxy (see Recipes)

8. 4× SDS-PAGE loading buffer (reducing) (see Recipes)

9. 10% Ammonium persulfate solution (see Recipes)

10. 10% SDS (see Recipes)

11. Resolving gel buffer (see Recipes)

12. Stacking gel buffer (see Recipes)

13. 10× SDS-PAGE running buffer (see Recipes)

14. 1× SDS-PAGE running buffer (see Recipes)

15. Western blotting transfer buffer (see Recipes)

16. Ponceau S staining solution (see Recipes)

17. 10× Tris-buffered saline, Tween-20 (TBS-T) (see Recipes)

18. Blocking solution (see Recipes)

19. 1:1,000 Streptavidin (HRP conjugate) solution (see Recipes)

20. ECL detection solution (see Recipes)

21. Resolving gel solution (see Recipes)

22. 4% stacking gel solution (see Recipes)

**Recipes**

**1. Cell culture medium**

**Table:** 

Reagent	Final concentration	Quantity or Volume	Quantity or Volume
FBS	10%	50 mL	56.8 mL
L-glutamine	1%	5 mL	5.7 mL
Penicillin-streptomycin	1%	5 mL	5.7 mL
DMEM	n/a	440 mL	500 mL

Keep at 4 °C.

**2. 0.1 M phosphate buffer, pH 7.4**

**Table:** 

Reagent	Final concentration	Quantity or Volume
Sodium dihydrogen phosphate	0.1 M	96.5 mL of 0.2 M stock solution
Disodium hydrogen phosphate	0.1 M	153.5 mL of 0.2 M stock solution
dH_2_O	n/a	200 mL
5 M HCl or 5 M NaOH	pH 7.4	As needed to adjust pH to 7.4
dH_2_O	n/a	Up to 500 mL

dH_2_O: deionized H_2_O

**Stock solutions**

**a. 0.2 M Sodium dihydrogen phosphate**

**Table:** 

Reagent	Final concentration	Quantity or Volume
NaH_2_PO_4_·H_2_O	0.2 M	27.598 g (MW: 137.99 g/mol)
dH_2_O	n/a	Up to 1 L

Store at room temperature.

**b. 0.2 M Disodium hydrogen phosphate**

**Table:** 

Reagent	Final concentration	Quantity or Volume
Na_2_HPO_4_·7H_2_O	0.2 M	53.614 g (MW: 268.07 g/mol)
dH_2_O	n/a	Up to 1 L

Store at room temperature.

**3. 5 M NaCl**

**Table:** 

Reagent	Final concentration	Quantity or Volume
NaCl	5 M	146.1 g NaCl (MW: 58.44 g/mol)
dH_2_O	n/a	Up to 500 mL

Store at room temperature.

**4. RIPA phosphate lysis buffer with protease and phosphatase inhibitors**

To the RIPA phosphate lysis buffer base, add protease and phosphatase inhibitors as indicated.

Protease/phosphatase inhibitors are prepared as stock solutions in distilled water, stored frozen, and added into the lysis buffer just prior to use as follows:

**Table:** 

Reagent	Stock solution concentration	Volume per 1 mL lysis buffer
Aprotinin	1 mg/mL	5 μL
Leupeptin	10 mg/mL	5 μL
Sodium fluoride (NaF)	0.2 M	5 μL
Sodium vanadate (Na_3_VO_4_)	0.2 M	5 μL
PMSF	0.1 M	15 μL
β-glycerophosphate	2 M	40 μL

Alternatively, dissolve one tablet of Complete Mini EDTA-free protease inhibitor cocktail in 10 mL of phosphate RIPA lysis buffer base. Aliquot and keep at -20 °C.

**Phosphate RIPA lysis buffer base (50 mL)**

**Table:** 

Reagent	Final concentration	Quantity or Volume
Phosphate buffer, pH 7.4	10 mM	5 mL of 0.1 M stock solution
NaCl	150 mM	1.5 mL of 5 M NaCl
NP-40	1%	0.714 g TERGITOL^TM^ solution (70% NP-40 in H_2_O)
DTT	5 mM	38.575 mg DTT
Sodium deoxycholate	0.1%	50 mg
dH_2_O	n/a	Up to 50 mL

The lysis buffer base is kept at 4 °C.

**5. 200 mM DTT**

**Table:** 

Reagent	Final concentration	Quantity or Volume
DTT	200 mM	15.43 mg (MW: 154.3 g/mol)
dH_2_0	n/a	Up to 500 μL

Prepare fresh just before use.

**6. 500 mM pPDA with 5 mM DTT**

**Table:** 

Reagent	Final concentration	Quantity or Volume
pPDA	500 mM	55.17 mg pPDA (MW: 108.14 g/mol)
DMSO	n/a	97.5 μL
DTT	200 mM	2.5 μL

For easier preparation of the reagent needed for multiple labeling reactions, it is recommended to weigh a larger amount and then adjust the volume of DMSO accordingly. For instance, if 200 mg of pPDA is placed in a 1.5 mL Eppendorf tube, 353.45 μL of DMSO should be added and vortexed until fully dissolved. Subsequently, for each 97.5 μL of this solution, 2.5 μL of freshly prepared 200 mM DTT should be added to create the final solution.

**7. 100 mM biotin-aminooxy**

**Table:** 

Reagent	Final concentration	Quantity or Volume
EZ-Link Alkoxyamine-PEG4-Biotin	100 mM	50.0 mg (MW: 154.3 g/mol)
DMSO	n/a	1,151 μL

Dissolve biotin-aminooxy in DMSO, make small, single-experiment aliquots, and store at -80 °C. Avoid multiple freeze-thaw cycles. This solution is stable for at least one year.

**8. 4× SDS-PAGE loading buffer (reducing)**

**Table:** 

Reagent	Final concentration	Quantity or Volume
0.5 M Tris-HCl, pH 6.8	0.25 M	5 mL
Glycerol	40% (v/v)	4 mL
SDS	8% (w/v)	0.8 g
2-Mercaptoethanol (2-ME)	4% (v/v)	0.4 mL
Bromophenol blue	0.01%	4 mg
dH_2_O	n/a	Up to 10 mL

**9. 10% Ammonium persulfate solution**

**Table:** 

Reagent	Final concentration	Quantity or Volume
Ammonium persulfate (APS)	10%	100.0 mg
dH_2_O	n/a	1 mL

Prepare fresh for each experiment.

**10. 10% SDS**

**Table:** 

Reagent	Final concentration	Quantity or Volume
Sodium dodecyl sulfate (SDS)	10%	10.0 g
dH_2_O	n/a	Up to 100 mL

Store at room temperature.

**11. Resolving gel buffer, 1.5 M Tris-HCl, pH 8.8**

**Table:** 

Reagent	Final concentration	Quantity or Volume
Tris base	1.5 M	181.7 g
dH_2_O	n/a	Up to 800 mL
HCl (37%)	pH 8.8	As needed to adjust pH to pH 8.8
dH_2_O	n/a	Up to 1 L

Store at 4 °C.

**12. Stacking gel buffer, 0.5 M Tris-HCl, pH 6.8**

**Table:** 

Reagent	Final concentration	Quantity or Volume
Tris base	0.5 M	60.6 g
dH_2_O	n/a	Up to 800 mL
HCl (37%)	pH 6.8	As needed to adjust pH to pH 6.8
dH_2_O	n/a	Up to 1 L

Store at 4 °C.

**13. 10× SDS-PAGE running buffer**

**Table:** 

Reagent	Final concentration	Quantity or Volume
Tris base	0.25 M	30.29 g (MW: 121.14 g/mol)
Glycine	1.92 M	144.13 g (MW: 75.07 g/mol)
SDS	0.1%	10 mL from 10% SDS
dH_2_O	n/a	1 L

Dissolve in 900 mL of dH_2_O, adjust to 1 L with dH_2_O, and store at room temperature. The buffer has a pH of around 8.3. Do not adjust its pH.

**14. 1× SDS-PAGE running buffer**

**Table:** 

Reagent	Final concentration	Quantity or Volume
10× SDS-PAGE running buffer	1×	100 mL
SDS	0.1%	10 mL of 10% SDS solution
dH_2_O	n/a	Up to 1 L

Keep at room temperature. If used infrequently, store at 4 °C.

**15. Western blotting transfer buffer**

**Table:** 

Reagent	Final concentration	Quantity or Volume
Tris base	25 mM	3.03 g (MW: 121,14 g/mol)
Glycine	0.192 M	14.4 g (MW: 75.07 g/mol)
Methanol	20%	200 mL
dH_2_O	n/a	Up to 1 L

Prepare on the day of transfer and cool down at -20 °C while running the SDS-PAGE.

**16. Ponceau S staining solution**

**Table:** 

Reagent	Final concentration	Quantity or Volume
Ponceau S	0.1% (wt/vol)	0.05 g
Glacial acetic acid	5% (v/v)	2.5 mL
dH_2_O	n/a	Up to 50 mL

Add ultrapure water up to 50 mL, wrap in aluminum foil, and store at room temperature.

**17. 10× Tris-buffered saline, Tween-20 (TBS-T)**

**Table:** 

Reagent	Final concentration	Quantity or Volume
Tris base	50 mM	6.05 g (MW: 121.14 g/mol)
NaCl	150 mM	8.76 g (MW: 58.44 g/mol)
HCl (37%)	pH 7.5	Adjust to pH 7.5
Tween-20	0.1 %	1 mL
dH_2_O	n/a	Up to 1 L

Add ultrapure water up to 800 mL and set the pH value up to 7.5. Add 1 mL of Tween-20 and add water up to 1,000 mL. Store at 4 °C.

**18. Blocking solution**

**Table:** 

Reagent	Final concentration	Quantity or Volume
Nonfat dry milk	5%	2.5 g
10× TBS-T	1×	5 mL
dH_2_O	n/a	Up to 50 mL

Prepare on the day of transfer and keep at 4 °C.

**19. 1:1,000 Streptavidin-HRP conjugate solution**

**Table:** 

Reagent	Final concentration	Quantity or Volume
Streptavidin-HRP	1‰	10 μL
10× TBS-T	1×	1 mL
dH_2_O	n/a	Up to 10 mL

Mix well. Prepare just prior to the incubation with the membrane.

**20. ECL detection solution**

**Table:** 

Reagent	Final concentration	Quantity or Volume
Pierce ECL Solution A	50%	2.0 mL
Pierce ECL Solution B	50%	2.0 mL

Mix the solutions just before use. For a typical size of the membrane in Mini-Protean Tetra Cell (9 × 7 cm), 4 mL of ECL detection solution is sufficient.

**21. Resolving gel solution**

**Table:** 

Gel density	Milli-Q water (mL)	30% acrylamide/ bis acrylamide (mL)	Resolving gel buffer (mL)	10% SDS (μL)
7%	5.1	2.3	2.5	100
8%	4.7	2.7	2.5	100
9%	4.4	3.0	2.5	100
10%	4.1	3.3	2.5	100
11%	3.7	3.7	2.5	100
12%	3.4	4.0	2.5	100

Add 50 µL of 10% APC and 5 µL of TEMED to each 10 mL of the resolving gel solution. Gently mix, then pour the mixture into the gel assembly.

**22. 4% stacking gel solution**

**Table:** 

Gel density	Milli-Q water (mL)	30% acrylamide/ bis acrylamide (mL)	Stacking gel buffer (mL)	10% SDS (μL)
4%	6.1	1.3	2.5	100

Add 50 µL of 10% APC and 5 µL of TEMED to each 10 mL of the stacking gel solution. Gently mix, then pour the mixture into the gel assembly.

**Laboratory supplies**

1. Parafilm^®^ M laboratory film (Amcor, catalog number: PM-996)

2. 0.1–10 μL pipette tips (Eppendorf, catalog number: 0030000811)

3. 0.5–20 μL pipette tips (Eppendorf, catalog number: 0030000854)

4. 5–200 μL pipette tips (Eppendorf, catalog number: 0030000889)

5. 50–1,000 μL pipette tips (Eppendorf, catalog number: 0030000927)

6. 1.5 mL tubes (Eppendorf, catalog number: 0030120086)

7. 2.0 mL tubes (Eppendorf, catalog number: 0030120094)

8. 15 mL tubes (Sarstedt AG & Co. KG, catalog number: 62.554.502)

9. 50 mL tubes (Greiner bio-one, catalog number: 227261)

10. 2 mL serological pipettes (Falcon, catalog number: 357507)

11. 5 mL serological pipettes (Falcon, catalog number: 357543)

12. 10 mL serological pipettes (Falcon, catalog number: 357551)

13. T75 cell culture flasks (Sarstedt AG & Co. KG, catalog number: 83.3911.002)

14. Mini Trans-Blot^®^ filter paper (Bio-Rad, catalog number: 1703932)

15. Immun-Blot^®^ LF PVDF membrane roll (Bio-Rad, catalog number: 162-0264)

## Equipment

1. Laminar air flow cabinet (Nüve, catalog number: LN090)

2. Cell incubator (Nüve, catalog number: EC160)

3. Inverted microscope (Olympus, model: CKX41)

4. Refrigerated laboratory centrifuge (Eppendorf, catalog number: 5920R)

5. Power supply (Bio-Rad, catalog number: 1645050)

6. Mini-PROTEAN 3 Cell (Bio-Rad, catalog numbers: 165-3301, 165-3302)

7. Mini trans-blot electrophoretic transfer cell (Bio-Rad, catalog number: 1703930)

8. Gel releaser for Mini-PROTEAN 3 (Bio-Rad, catalog number: 1653320)

9. Refrigerated microcentrifuge (Eppendorf, catalog number: 5427R)

10. 0.5–10 μL pipette (Eppendorf Research plus, catalog number: 3123000020)

11. 2–20 μL pipette (Eppendorf Research plus, catalog number: 3123000039)

12. 20–200 μL pipette (Eppendorf Research plus, catalog number: 3123000055)

13. 100–1,000 μL pipette (Eppendorf Research plus, catalog number: 3123000063)

14. Pipette filler (MediLab Tech, catalog number: 7013100200)

15. Eppendorf ThermoMixer^®^ C (Eppendorf, catalog number: 5382000023)

16. 3D shaker (Cleaver Scientific, catalog number: MW-23)

17. Vortex shaker (IKA, catalog number: 10142902)

18. Laboratory balance (Radweg, catalog number: PS750.R1)

19. pH meter (HACH, catalog number: HQ30D53000000)

20. BioDrop^TM^ μLite+ (VWR, catalog number: 80-3006-55)

21. ChemiDoc^TM^ MP imaging system (Bio-Rad, catalog number: 12003154)

## Software and datasets

1. Image Lab software (Bio-Rad)

## Procedure

**A. Cell culture**

1. Culture A549 cells in cell culture medium (see Recipes) in a humidified atmosphere with 5% CO_2_ at 37 °C until they reach 80% confluence.

**B. Cell lysis and protein quantification**

1. Remove the cell culture medium and rinse the flask with 10 mL of ice-cold PBS. Discard the PBS, add 1.5 mL of Trypsin EDTA, and incubate at 37 °C for about 5 min.

2. Swirl the flask and gently tap to verify that cells have begun to detach.

3. Add 9 mL of cell culture medium to neutralize the trypsin and collect detached cells.

4. Centrifuge the cell suspension at 300× *g* for 10 min at 4 °C.

5. Discard the supernatant and wash the cells 2× with PBS as described above.

6. Add 0.1 mL of lysis buffer (see Recipes) per 10^7^ cells.

7. Incubate on ice for 30 min while vortexing for 30 s every 10 min.

8. Centrifuge the lysate at 15,000× *g* for 15 min at 4 °C.

9. Transfer the supernatant into a new 1.5 mL microcentrifuge tube.

10. Determine the protein concentration. In this work, we did it through OD measurement at 280 nm on a BioDrop^TM^ μLite+, but it could also be done using a BCA assay from Thermo Fisher Scientific.

11. Adjust the protein concentration to about 5 mg/mL using the RIPA phosphate lysis buffer with protease and phosphatase inhibitors.

**C. Carbonyl labeling**

1. Set up the labeling reaction by combining the following in the 1.5 mL (or 2.0 mL) Eppendorf tube:

80 μL of cell lysate

10 μL of 100 mM aminooxy-biotin (see Recipes)

10 μL of 500 mM pPDA with 5 mM DTT (see Recipes)

2. Set aside a negative control tube containing 80 μL of cell lysate and 20 μL of RIPA phosphate lysis buffer with protease and phosphatase inhibitors (no aminooxy-biotin or pPDA/DTT added).

3. Incubate the samples in a thermomixer at 25 °C and 500 rpm for 3 h.

4. After incubation, add 33.3 μL of 4× loading buffer (see Recipes) to each sample.

5. Denature the samples by heating them at 95 °C for 5 min.

6. Use immediately for the Oxime blot analysis or store at -20 °C until further analysis.

**D. Oxime blot analysis**

**D1. SDS-PAGE**

1. Assemble the glass plates for gel casting and ensure that the bottoms are even.

2. Insert into the casting stand and check for leaks by adding a small amount of dH_2_O.

3. Pour out the liquid and dry the space.

4. Prepare a resolving gel (see Recipes). Stir briefly and gently pipette approximately 5.5 mL (1.5 mm gel) of the solution. Make sure to have about 5 mm from the bottom of the comb to the resolving gel.

5. Add 1 mL of isopropanol on top of the gel and wait until it polymerizes.

6. Decant the isopropanol and rinse with the dH_2_O to remove any residual isopropanol. Drain thoroughly and wipe the water from the plates before pouring the stacking gel.

7. Pour the 4% stacking gel (see Recipes). Insert the combs at an angle to avoid trapping any bubbles below the bottom of the well.

8. Wait until the stacking gel polymerizes (about 30 min). It is convenient to prepare the gels in advance. When kept in moist paper tissue in a tightly closed container at 4 °C, they can be used for several days.

9. Prepare 1× SDS-PAGE running buffer (see Recipes).

10. Remove the comb and wash the wells with 1× SDS-PAGE running buffer 2–3 times, draining in between the washes.

11. Assemble the electrode assembly cassette, fill it with the running buffer, and ensure it does not leak.

12. Load the prestained markers (3–4 μL) and samples (10–30 μL, depending on the protein content) into the wells.

13. Add running buffer into the lower buffer chamber until the lower end of the gel plates is about 1 cm immersed in the buffer.

14. Initiate the electrophoresis at 100 V until the samples cross from the stacking to the resolving gel.

15. Increase the voltage to 150 V and continue protein separation until the front dye reaches the bottom of the gel.

16. Disassemble the electrode assembly and separate the glass plates.

17. Remove the stacking from the resolving gel using a gel spatula or glass plate.

18. Use the gels to visualize protein separation by Coomassie blue staining or for protein transfers and subsequent detection of carbonylated proteins by Oxime blot.

**D2. Coomassie blue staining**

**Figure 1.**
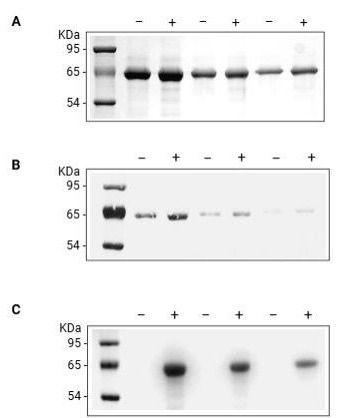
Spontaneous carbonylation of BSA. BSA dissolved at 5 mg/mL in dH_2_O was incubated with either aminooxy-biotin and pPDA catalyst (+) or an equal volume of 10 mM phosphate buffer (-). Equal quantities of unlabeled (-) and labeled (+) BSA (10, 5, and 2.5 μg/lane, respectively) were resolved in adjacent lanes by SDS-PAGE and analyzed by (A) Coomassie blue staining for the total protein or (B) Ponceau S staining to confirm the protein transfer to the PVDF membrane and (C) Oxime blot to analyze the BSA carbonylation.

**Figure 2.**
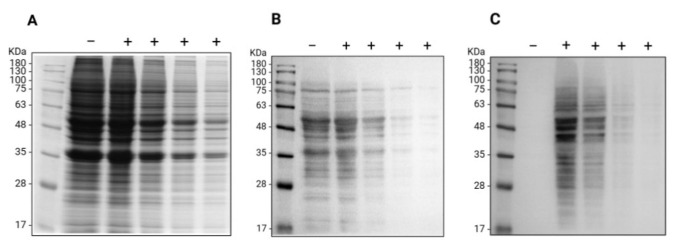
Oxime blot of A549 cell lysate proteins. Proteins extracted from A549 cells were derivatized (+) with aminooxy-biotin in the presence of the pPDA catalyst or used as a negative control (-) for samples treated with an equal volume of the phosphate buffer. A decreasing amount of total proteins (from 46 to 5.75 μg/lane) were resolved by SDS-PAGE proteins and analyzed by (A) Coomassie blue staining for the total protein pattern or following the transfer to the PVDF membrane by (B) Ponceau S staining to confirm the protein transfer and (C) Oxime blot to analyze the carbonylation of endogenous A549 proteins.

1. Mix the InstantBlue^®^ Coomassie protein stain by gently inverting the bottle a few times immediately before use.

2. Completely immerse the gel in about 20 mL of InstantBlue^®^ stain and incubate at room temperature with gentle shaking until the required intensity is achieved (usually 30–60 min).

3. Briefly rinse with dH_2_O.

4. Image the stained gel with an imaging system such as ChemiDoc^TM^ MP imaging system and analyze using software such as Image Lab (Figures 1A, 2A, and Figure S1A Extended data).

**D3. Oxime blot analysis**

1. Activate the PVDF membrane by soaking it in 100% methanol for 1 min.

2. Rinse with dH_2_O for 2–3 min.

3. Incubate in transfer buffer until use.

4. Immerse sponge pads in the previously cooled transfer buffer to facilitate the removal of the bubbles.

5. Assemble the transfer sandwich. The cathode side of the transfer sandwich on the Bio-Rad device is marked in black. The correct order from the black transfer sandwich sheet is as follows: sponge pad, filter paper, gel, PVDF membrane, filter paper, sponge pad from cathode to anode. Remove bubbles between the gel and membrane using a round object such as a part of a 10 or 50-mL plastic pipette.

6. Place the sandwich in the electrode module, observing the correct orientation relative to the electrical current direction [black, black transfer sandwich sheet closer to the black (cathode) side of the electrode module].

7. Pour the cold transfer buffer into the transfer tank with the top level just below the top of the electrode module.

8. Place the transfer tank in an insulated Styrofoam box and surround it with ice and ice-cold water. Sporadically add ice to the mix to ensure efficient cooling.

9. Transfer proteins from the gel to the PVDF membrane at 400 mA for 2 h (for two gels).

10. Check that proteins are transferred by staining the blotted membrane with Ponceau S solution (see Recipes).

11. Immerse the membrane in the Ponceau S solution with gentle shaking for 5–15 min (until proteins are easily visible).

12. Decant the stain in the original container, since the dye can be reused for several weeks.

13. Remove the bulk of the stain with 2–3 quick rinses with dH_2_O while shaking.

14. De-stain the membrane by incubating it for 5 min in dH_2_O on a 3D orbital shaker at 30 rpm.

15. Take images on the imaging system if required for record keeping (Figures 1B and 2B).

16. Block the nonspecific binding of the aminooxy-biotin by incubating the membrane in blocking solution (see Recipes) for 1 h at room temperature with gentle shaking on a 3D orbital shaker at 30 rpm.

**Pause point:** Blocking could be extended overnight if convenient. Wash the membrane twice with 1× TBS-T (see Recipes) for 5 min each.

17. Wash the membrane twice with 1× TBS-T (see Recipes) for 5 min each.

18. Prepare 10 mL of 1:1,000 Streptavidin (HRP conjugate) solution (see Recipes) for each membrane.

19. Incubate for 1 h at room temperature with gentle shaking on a 3D orbital shaker at 30 rpm.

20. Wash the membrane 3× with 15–20 mL of 1× TBS-T (see Recipes) for 5–10 min each.

21. Prepare 4 mL of ECL detection solution (see Recipes) for each membrane and use immediately.

22. Apply the detection solution to the membrane and take the image on the imaging system (Figures 1C, 2C, and Figure S1B Extended data).

## Validation of protocol

This protocol was developed and validated in the research article by Ladouce et al. [16] (see Figures 2A and 3C).

## General notes and troubleshooting

**General notes**

1. This protocol describes labeling carbonylated proteins with an aminooxy-biotin probe in the presence of a pPDA catalyst. The labeling reaction is very reproducible.

2. The method is suitable for carbonylated protein labeling over a broad range of concentrations (from immunoprecipitates to >5 mg/mL) and sample types. It can detect the spontaneous carbonylation of BSA in the low ng/lane range.

3. It is expected that the same reaction could be used to detect carbonylated proteins by ELISA, but this has not been formally tested.

4. This protocol is particularly well suited for detecting specific carbonylated proteins after their immunoprecipitation, labeling with an aminooxy-biotin in the presence of a pPDA on the beads, and boiling for 5 min at 95 °C in 1× SDS-PAGE loading buffer.

5. Aminooxy-biotin is stable for at least one year without noticeable loss of activity when kept aliquoted at -80 °C to avoid repeated freeze-thaw cycles.

**Troubleshooting**

Problem 1: Low level of protein on the membrane.

Possible causes: Low quantity of protein used for the SDS-PAGE or poor protein transfer to the membrane.

Solution: Check the protein quantity and resolution of SDS-PAGE separation by staining the gel with Coomassie blue and protein transfer on the membrane by Ponceau S staining. For the vast majority of proteins, the described transfer conditions will work fine. However, for very large or small proteins, transfer conditions may need to be optimized.

Problem 2: Poor resolution of carbonylated proteins in cell lysates.

Possible causes: Poor cell lysate quality or low protein amount used. Too much mechanical stress is applied.

Solution: Start with the recommended quantity of proteins for labeling and use aminooxy-biotin derivatized BSA as a positive control. Once the cell pellet is dispersed by adding lysis buffer and pipetting, vortex the samples as described during cell lysis.

## Supplementary information

The following supporting information can be downloaded here:

1. Figure S1. Extended data
